# The design of synthetic gene circuits in plants: new components, old challenges

**DOI:** 10.1093/jxb/erad167

**Published:** 2023-05-19

**Authors:** Marta Vazquez-Vilar, Sara Selma, Diego Orzaez

**Affiliations:** Instituto de Biología Molecular y Celular de Plantas (IBMCP), Consejo Superior de Investigaciones Científicas (CSIC), Universitat Politècnica de Valéncia, Camino de Vera s/n, 46022 Valencia, Spain; Instituto de Biología Molecular y Celular de Plantas (IBMCP), Consejo Superior de Investigaciones Científicas (CSIC), Universitat Politècnica de Valéncia, Camino de Vera s/n, 46022 Valencia, Spain; Instituto de Biología Molecular y Celular de Plantas (IBMCP), Consejo Superior de Investigaciones Científicas (CSIC), Universitat Politècnica de Valéncia, Camino de Vera s/n, 46022 Valencia, Spain; Centre for Research in Agricultural Genomics (CRAG), Spain

**Keywords:** Actuator, CRISPRa, logic gates, plant synthetic biology, processor, sensor, synthetic gene circuits

## Abstract

The fascination produced by the possibility of engineering plants with augmented capabilities has accompanied plant biotechnology since its origins. This prospect has become even more relevant in present times under the pressure imposed by climate change and population growth. Today’s plant biotechnologists approach this challenge with the tools of synthetic biology, which facilitate the assembly of synthetic gene circuits (SGCs) from their modular components. Transcriptional SGCs take environmental or endogenous inputs and operate them using transcriptional signals in ways that do not necessarily occur in nature, generating new physiological outputs. Many genetic components have been developed over the years that can be employed in the design and construction of plant SGCs. This review aims to provide an updated view of the components available, proposing a general scheme that facilitates the classification of circuit components in sensor, processor, and actuator modules. Following this analogy, we review the latest advances in the design of SGCs and discuss the main challenges ahead.

## Teaser

This review aims to provide a framework for categorizing the different components of synthetic gene circuits, thus facilitating the exchange of DNA parts and information in plant synthetic biology.

## Introduction

Modern societies are avid consumers of bioproducts, from the foods necessary to sustain a world population in continuous growth to the most complex biopharmaceuticals required to maintain or even extend the life expectancy of that same population. Not surprisingly, one of the great challenges for the future will consist of responding to these requirements for bioproducts with increasingly efficient, but at the same time, sustainable production systems. Genetic engineering and, more recently, synthetic biology (SynBio) are among the technologies that can contribute more to this challenge.

Plants are called upon to play a fundamental role in the future of bioproduction sustainability, in what refers not only to basic food and feed supply but also to a growing plethora of new bioproducts that society demands, from construction and packaging materials to agrochemicals or biopharmaceuticals. A recent study showed that 83% of the biomass generated on the planet is of plant origin (Bar-On *et al.*, 2018), indicating that the biosynthetic capacity of plants is unmatched by any other evolutionary group. From a biotechnological point of view, the preponderance of plants as global bioproducers confirms that the photoautotroph plant chassis provides indisputable advantages in terms of sustainability to lead the biofactories field in the future. However, beyond food production, it turns out that the current manufacturing trends point towards traditional microbial/cellular fermenters instead of plants as preferred industrial biofactories. One of the main reasons for that is that process control technology is much more developed in the case of biofermenters. Plant-based production systems are subjected to strong seasonal/batch fluctuations that are poorly compatible with the uniform and reliable production demanded by the industry. Furthermore, except for *Agrobacterium*-mediated transient expression-based technology (Krenek *et al.*, 2015), mechanisms to reliably modify or enrich the chemical composition of the plant biomass in a controllable manner are poorly developed.

From all the above, it can be inferred that a more effective control of the plant’s chassis would enable a more efficient exploitation of its innate capacities. For this, new genetic control systems need to be implemented that buffer external changes (such as those derived from climate change), or that enable new regulatory capacities and agrochemical-induced gene expression. Genetic control in living cells can be implemented with the introduction of new synthetic gene circuits (SGCs). An SGC is a rationally designed group of genes (including their associated regulatory sequences), that drive new input–output operations using RNA transcripts as the physical support for the transmission of information. In recent years, SynBio has made very important contributions to microbial bioproduction by designing new SGCs that increase the control capabilities of the system. Equivalently, the next challenge in plant biotechnology will involve the addition of new genetic control mechanisms to plant-based production. The genetic complexity of plants, the lack of operons in the functional organization of their genomes, and their multicellular nature make this task especially difficult. However, although the technological challenge is great, the potential advantages make it worthwhile.

In this review, we will analyse the genetic components required to build SGCs in plants, preferably following a SynBio-oriented framework. We will employ a general scheme earlier proposed by Qi and co-workers that covers the most common circuit architectures (H. Qi *et al*., 2013; Gao *et al.*, 2019; Moore *et al*., 2022). This scheme is based on electronic circuits and therefore the nomenclature employed includes terms borrowed from this discipline. The SynBio jargon is rich in analogies with electronics, sometimes at the cost of more common biology terms, raising some criticism (de Lorenzo and Danchin, 2008; McLeod and Nerlich, 2017). We believe that analogies, if used correctly, help bioengineers in the process of abstraction of function, one of the foundational principles of SynBio. Therefore, in this review, we will take the liberty of employing many electronic analogies to describe some biological components.

## Dissecting synthetic transcriptional gene circuits

**Fig. 1.**
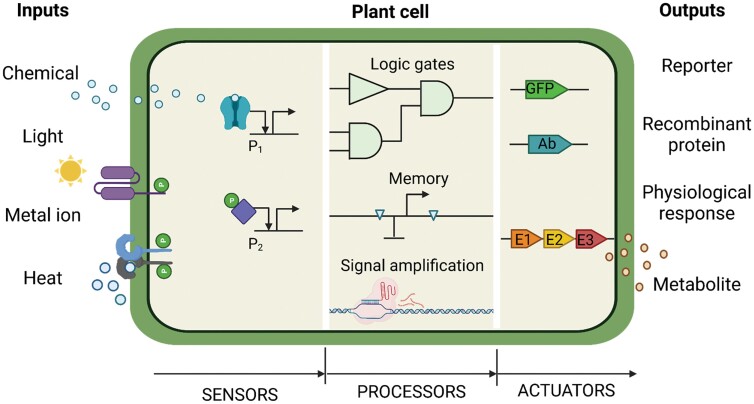
The components of a synthetic gene circuit are classified into sensors, processors, and actuators. Sensors take non-transcriptional inputs and convert them to transcriptional outputs. Processors transform a transcriptional input into a transcriptional output of a different kind. Actuators convert a transcriptional input into a non-transcriptional output.

The architecture of naturally occurring, as well as engineered, circuits typically comprises three functional modules, namely sensors, processors, and actuators (Fig. 1) (De Las Heras *et al.*, 2010; H. Qi *et al*., 2013). A sensor is defined as the module that inputs a non-transcriptional signal and produces a transcriptional output. Sensors are transcriptionally connected with an operator, which transforms a transcriptional input into a transcriptional output of a different kind. Operators can, for instance, amplify input signals, distribute them to many downstream elements, store them as memories of events, or integrate them following Boolean logic, among many other possible operations. The transcriptional output signals produced by operators are then input by actuators, the last functional module in a canonical SGC, to convert them into non-transcriptional outputs. Enzymes are the prototypical actuators in plant cells. Bioengineers often use reporter genes (fluorescent proteins, coloured enzymatic reactions, or luciferases) as model actuators to easily detect and quantify SGC outputs. Gene circuit-like structures do occur naturally in plant cells, although usually each circuit component does not operate in isolation but is influenced by many components of other circuit-like structures, creating a complex interaction network (Hyun *et al.*, 2019). In a recurrent theme, individual plant gene circuits are connected to each other by second messengers and/or phytohormones synthesized and/or released by actuators in circuit 1 and detected by sensors in circuit 2. One of the main challenges that plant bioengineers face is to design ‘orthogonal’ circuits, namely SGCs whose components show minimal interactions with the rest of the components in the cell (often referred to as ‘the chassis’ in SynBio jargon). A general strategy to maximize orthogonality consists of the use of non-plant genetic elements (e.g. bacterial or mammalian gene parts) to create plant SGCs. Similarly, plant genes are sometimes employed to build orthogonal circuits in mammalian systems. A very successful example of this practice is the use of plant red/far-red or UV light receptors for the design of orthogonal optogenetic sensors in mammalian gene circuits (Müller *et al.*, 2013, 2014a).

## Enabling tools and technologies for gene circuit design and construction in plants

**Fig. 2.**
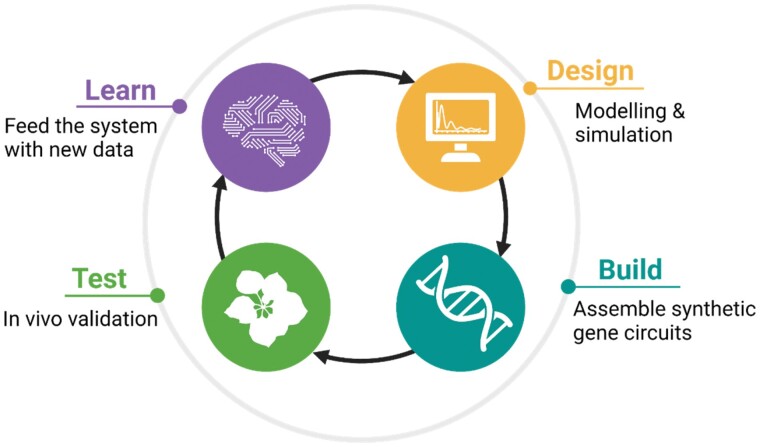
The design–build–test–learn (DBTL) cycle is used for optimization of synthetic gene circuits. Computer-assisted design is poorly developed in plants. The building step has advanced in the last decade with modular cloning systems. Testing is nowadays the main experimental bottleneck, partially circumvented by transient expression systems. Key design hints (minimal promoter activities, positional effects, etc.) are learnt in each cycle which are implemented in new iterations.

Equipping plants with reliable SGCs is nowadays still a major technological challenge, yet the adoption of new SynBio approaches is paving the way. SynBio imprints a working methodology characterized by the implementation of so-called design–build–test–learn (DBTL) cycles (Fig. 2). This implies the realization that one-shot approaches are unlikely to fully work at a first attempt and, instead, initial SGC designs need to be tested and refined in iterative cycles, so that each iteration interrogates the cell about the adequacy of the current design and provides engineers with the clues for improving the design in the next iteration. Engineering through DBTL cycles requires agile and high-throughput experimental setups to speed up the process.

The implementation of computer-aided design (CAD) tools, which assign genetic parts and define their order following their expected functional roles, is still very limited in plant SynBio. Despite some interesting attempts to incorporate plant-specific grammar into available CAD tools such as GenoCAD (Coll *et al.*, 2016; Lukan *et al.*, 2022), circuit design in plants remains a rather handmade, unassisted process. This is despite the increasing number of collections of DNA parts available for plants, and the efforts in providing quantitative characterizations to those parts (Schaumberg *et al.*, 2016; Vazquez-Vilar *et al.*, 2017). In contrast, circuit building at the DNA level has advanced rapidly in the last decade thanks to the general adoption of highly efficient modular cloning methods (for a review, see Patron, 2014), and the increasing affordability of gene synthesis, to the extent that today it can be affirmed that DNA assembly is no longer a limiting step. Instead, the experimental bottleneck has been transferred to the ability to test new constructs *in vivo* in a high-throughput manner. For this, transient expression methods have become the option of choice, given the time and effort required for generating stable plants. Among transient systems, protoplast transfection and *Nicotiana benthamiana* agroinfiltration are the two most widely used methodologies. Protoplast transfection allows high-throughput analysis, and it has been the method of choice for quantitative part characterization (Schaumberg *et al.*, 2016), as well as for testing optogenetic circuits (Ochoa-Fernandez *et al.*, 2020), among many other examples. A possible limitation of the protoplast system is the harsh experimental conditions to which cells are subjected during the process, which can make circuit behaviour differ from that of more physiological conditions in stable plants. Agroinfiltration of *Nicotiana* leaves employs more physiological conditions, and it is the method of choice for testing gene constructs for metabolic engineering, as it allows easy scale-up for metabolite analysis (Molina-Hidalgo *et al.*, 2021). It also shows remarkable co-transformation efficiencies, reducing DNA assembly requirements for the analysis of multigenic constructs and facilitating combinatorial experiments. The main drawbacks are the lower throughput capacity and the fact that it is mainly limited to a single species, *N. benthamiana*, whose unmatched efficiency as a host for *Agrobacterium*-mediated transient expression is only partially explained (Bally *et al.*, 2018).

The enabling technologies described above, next to the progressive incorporation of the principles of modularity, standardization, and abstraction of function to the experimental strategies implemented in plant genetic engineering, are impacting our ability to program new gene functions in plants. In addition, plant bioengineers count nowadays with a large palette of genetic building blocks developed over the years with which they can create multiple circuits with a large diversity of functions. Next, we will review some of these building blocks, with special attention to those tested successfully in plant systems.

## Sensor modules

As mentioned before, sensor modules input environmental or intracellular non-transcriptional signals and convert them to transcriptional outputs. In recent years, there has been an intense effort in the development of molecular sensors for the perception of both endogenous and exogenous signals. Sensors can be classified according to the nature of their input signal as physical and chemical sensors, the latter being the most developed. Among the chemical sensors, phytohormone sensors are of special interest for molecular physiological research. One of the best characterized is the auxin sensor. Initially developed in yeast (Havens *et al.*, 2012), it was later transferred to plants (Brunoud *et al.*, 2012), allowing the monitoring, through a reporter system, of the auxin levels in the plant and their distribution. Currently, several optimized versions of these sensors have been developed to respond to different auxin concentrations, offering alternative monitoring outputs (Wend *et al.*, 2013; Liao *et al.*, 2015; Lieberman-Lazarovich *et al.*, 2019; Herud-Sikimić *et al.*, 2021). In parallel, a cytokinin sensor was developed, showing green fluorescent protein (GFP) patterns that reflect the signalling network of cytokinins in plants. This sensor, as well as its employment in *Arabidopsis thaliana*, was correctly adapted to maize (D’Agostino *et al.*, 2000; Zürcher *et al.*, 2013). The jasmonic acid (JA) sensors coupled to expression of a fluorescent protein, such as the so-called JAI3–GFP (Chini *et al.*, 2007) and Jas9–VENUS (Larrieu *et al.*, 2015), have been developed to visualize the dynamic changes in JA as a stress response in plants. Other examples of phytohormone sensors are the ABAleons (Waadt *et al.*, 2014), ABACUS (Jones *et al.*, 2014), and SNACS (Zhang *et al.*, 2020) sensors, which, in the presence of ABA (abscisic acid), can generate a change in bioluminescence emission and monitor the levels of this hormone. Also, a great variety of ethylene sensors have been developed to respond to the presence of this phytohormone in plants, such as the EIN3–GFP (Guo and Ecker, 2003), EIL1–GFP (An *et al.*, 2010), GFP–EBF2 3ʹ-untranslated region (UTR) (Merchante *et al.*, 2015), GFP–6×EPU (Li *et al.*, 2015), EBS:GUS (β-glucuronidase) sensors (Stepanova *et al.*, 2007), and, more recently, the AEP sensor (Vong *et al.*, 2019), which is an ethylene-sensing bioimaging technique based on the artificial metalloenzyme action. Other approaches are focused on the detection of exogenous (Silverstone *et al.*, 2001) or endogenous (Rizza *et al.*, 2017) gibberellin levels in plants, salicylic acid (SA) monitoring (Mou *et al.*, 2003), or spatiotemporal brassinosteroid determination (Chaiwanon and Wang, 2015).

Beyond hormone sensors, able to perceive the endogenous state of the plant, other types of sensors capable of detecting chemical changes in the environment are essential components in SGCs. Chemical receptors were the first sensors of exogenous stimuli adapted to plants, and immediately became essential components for inducible gene expression. The tetracycline sensor system, for instance, is a de-repressor of transcription. In the absence of tetracycline, the bacterial tetracycline repressor (TetR) binds to the tet operator and blocks the transcription of the coupled gene. This system was adapted to plants and employed for controlling the expression of genes in tobacco, tomato, and potato (Gatz *et al.*, 1992; Weinmann *et al.*, 1994; Bortesi *et al.*, 2012). In contrast to TetR, most chemical sensors act by activating transcription. This is the case of the sensors of the family of steroids widely employed in plants, which comprise the glucocorticoid sensor (Aoyama and Chua, 1997; Samalova *et al.*, 2005), the estrogen sensor (Bruce *et al.*, 2000; Okuzaki *et al.*, 2011), and the ecdysone sensor (You *et al.*, 2006). In these systems, the transcription signal remains off until the hormone ligand is bound to the steroid receptor allowing its translocation to the nucleus. The steroid receptor is engineered to contain a DNA-binding domain and a regulation domain to induce the transcription of a target gene. Other sensors based on the specific recognition of small chemical molecules more suitable to be employed in agriculture have also been developed, such as the copper-, ethanol-, and insecticide-induced systems. The system based on copper recognizes higher levels of this element than the endogenous levels present in the plant, generating a conformational change of the copper-responsive factor CUP2 fused to an activation domain that results in transcriptional activation of the gene driven by the CBS operator (Saijo and Nagasawa, 2014; Garcia-Perez *et al.*, 2022). The ethanol-induced system is based on the fungal protein AlcR that responds to ethanol and generates activation of the gene that is downstream of the *pAlcA* promoter (Caddick *et al.*, 1998; Li *et al.*, 2005). The insecticide-induced systems are based on the specific ligand binding on ecdysone receptors (EcRs) from insects. This sensor was optimized to recognize synthetic ecdysone agonists to regulate target gene expression (Padidam *et al.*, 2003; Koo *et al.*, 2004).

Sensors that respond to physical stimuli, such as light, were developed in recent years in plants. Optogenetic sensors are based on proteins that suffer structural changes when irradiated with light of a specific wavelength (Christie and Zurbriggen, 2021). The first optogenetic sensor to be efficiently employed in *N. benthamiana* and *A. thaliana* was based on the PHYB–PIF phytochrome interaction that induces transcription by irradiation with red light (Müller *et al.*, 2014b; Ochoa-Fernandez *et al.*, 2016). To get tighter control of gene expression, this approach was combined with the optogenetic system based on the LOV transcription factor (Pudasaini *et al.*, 2015). The generation of a synthetic bipartite promoter, controlled by LOV fused to a repressor domain and by PHYB fused to an activator domain, leads to a repressed state of the target gene in white light and a specific activation under monochromatic red light (Ochoa-Fernandez *et al.*, 2020). Other optogenetic approaches involve the cryptochrome CRY2-CIB1 sensor that transcriptionally activates the target gene by the action of blue light (Duan *et al.*, 2017) or the CarH photoreceptor system that, in the presence of adenosylcobalamin cofactor (AdoB12), activates transcription with green light (Chatelle *et al.*, 2018). Successful examples of optogenetic circuits in plants are, for example, the manipulation of auxin regulatory networks through the red light-inducible system (Müller *et al.*, 2014b), and the increment of the biomass production in *A. thaliana* achieved through the blue light-induced K^+^ channels. This system, named BLINK1, controls stomatal movements and the K^+^ uptake in leaves, generating an increase in guard cell volume and turgor and reducing water requirements (Papanatsiou *et al.*, 2019).

Recently, a general method to generate biosensors for user-defined molecules has been developed. This approach uses PYR1 (Pyrabactin Resistance 1), a plant ABA receptor with a malleable ligand-binding pocket, to engineer new sense–response functions (Beltrán *et al.*, 2022). Using this new approach, 21 new sensors for a range of small molecules were developed and readily ported to transcriptional circuits. The new sensing molecules include synthetic cannabinoids and organophosphates. Rapid biosensor development using plant hormone receptors as reprogrammable scaffolds opens up new possibilities for SGC development.

The collection of sensors that respond to different stimuli keeps growing (Schwarzländer and Zurbriggen, 2021). In the context of the development of SGCs, sensors represent the first step in the control of gene expression. However, the direct coupling of a sensor to an actuator through a basic processor that offers only unsustained ‘on’ (identity function) or ‘off’ (negation function) transcriptional responses, as happens in most traditional inducible systems available for plants, is not sufficient to integrate the complex responses required in innovative plant breeding. It is necessary to develop new genetic modules that operate as processors, therefore increasing the range of synthetic transcriptional responses that can be generated in the plant chassis.

## Building blocks for plant transcriptional processors

The simplest way to operate the transcription of a specific set of genes consists of the ectopic expression of natural transcriptional factors (TFs) (Zhang, 2003; Hong, 2016). These TFs can be placed under the control of regulated promoters, therefore the promoter-specified inputs (sensor) are connected to a cascade of TF-targeted activated/repressed genes as output (Li *et al.*, 2013; Petolino and Davies, 2013). A limitation of this scheme is that it does not allow free selection of the output response, as the collection of target genes is restricted by the DNA binding specificities of the natural TFs employed. Chimeric TFs offer more versatility to this basic scheme. They usually comprise a DNA-binding domain, which specifically binds a DNA operator in the promoter of target genes, and a transactivator/repressor domain (TAD/TRD). One of the first described TADs was derived from yeast GAL4 TF, and served to demonstrate the orthogonality of TADs and their potential to operate in various species and genomic contexts (Hope and Struhl, 1986; Keegan *et al.*, 1986). The modular nature of many TFs led to the identification of powerful viral TADs, such as the VP16 domain of the herpes simplex virus (Campbell *et al.*, 1984; Carey *et al.*, 1990), which proved to be a powerful activator also in plants (Moore *et al*., 1998; Schwechheimer *et al.*, 1998). Additionally, this domain offered the possibility of increasing its transcriptional activation potential through the fusion of several repetitions in tandem, originating the synthetic activation domains VP64, VP128, etc. These domains offered a greater activation range of target genes in different biological systems (Ordiz *et al.*, 2002; Li *et al.*, 2017). Modular regulatory domains of plant origin have also been identified. Two examples of plant TADs are the ERF2 and the EDLL domains, both from the Ethylene Response Factor (ERF) family (Tiwari *et al.*, 2012; Li *et al.*, 2013). The ERF family also includes proteins with identified TRDs, such as the EAR (Ethylene-responsive element-binding factor-associated amphiphilic repression) motifs, with which it has been possible to obtain efficient transcriptional repression. A remarkable example is ERF3 (Ohta *et al.*, 2001; Tiwari *et al.*, 2004) from which the widely used TRDs, SRDX, and BRD are derived (Hiratsu *et al.*, 2003; Ikeda and Ohme-Takagi, 2009).

### Programmable transcriptional regulators (PTRs) as versatile elements for the design of genetic processors

**Fig. 3.**
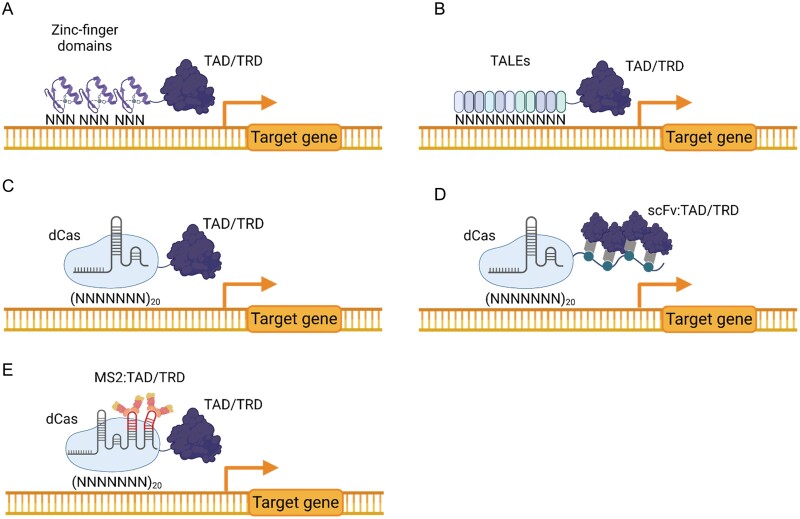
Programmable transcriptional regulators (PTRs). (A) Zinc finger transcriptional regulators. Three to six zinc finger DNA-binding domains (recognizing three nucleotides each) are fused to a transcriptional activator or a transcriptional repressor domain (TAD/TRD). (B) Transcription activator-like effector (TALE) transcriptional regulators. Multiple TALE monomers (each recognizing one nucleotide) are linked in tandem to recognize the desired DNA sequence. The TALE array is translationally fused to a TAD/TRD. (C) dCas-based transcription factors. The RNA-guided dCas protein is translationally fused to a TAD/TRD. (D) dCas-SunTag transcriptional regulators. The RNA-guided dCas protein is translationally fused to a multiepitope tail with multiple binding sites for TADs/TRDs. (E) dCas/scRNA transcriptional regulators. The RNA-guided dCas protein is translationally fused to a TAD/TRD. The sgRNA includes aptamers with binding sites for the MS2 coat protein. The MS2 coat protein is translationally fused to a TAD/TRD.

The main drawback of chimeric TFs is that their hardwired DNA specificity limits the choice of target genes, as these should obligatorily contain the cognate *cis* DNA operator. The possibility of programming the specificity of transcriptional regulators arrived with the discovery of artificial zinc fingers (ZFs) and transcription activator-like effectors (TALEs). These tools offered the option of creating customizable TFs. The artificial ZFs (Fig. 3A) are customizable DNA-binding domains that typically recognize 3–6 nucleotide triplets. Artificial ZFs were designed initially for targeted mutagenesis, producing double-strand DNA breaks with the fusion of FokI nucleases (Durai *et al.*, 2005). Later, the technology evolved to artificial TFs by including translational fusions to transcriptional regulator domains or epigenetic effectors (Shrestha *et al.*, 2018). Likewise, the engineered TALEs share many similarities in operation and structure with ZFs but offer a higher specificity. TALEs are proteins from bacteria of the genus *Xanthomonas*, which participate in plant infection mechanisms by promoting the expression of host genes. TALEs (Fig. 3B) consist of a specific and customizable DNA-binding domain comprising tandem repeat arrays of amino acids, which can recognize a specific DNA target sequence (Boch *et al.*, 2009; Moore *et al*., 2014). As in the case of ZF, the action mechanism of TALEs requires a new design of the protein for each target sequence, which makes them efficient but labour-intensive tools.

In plants, several works describe ZFs and TALEs being employed as artificial TFs, thus enabling programmable gene regulation. The first ZF examples targeted the *APETALA3* gene in *A. thaliana*. The VP64 TAD and the mSin3 interaction domain TRD were fused to an *APETALA3* ZF, yielding the expected transcription changes and generating altered floral patterns (Guan *et al.*, 2002). In parallel, engineered TALEs were proven as efficient customizable transcriptional regulators in plants. Interesting examples are the regulation of EGL3 and KNAT endogenous genes in *A. thaliana* (Morbitzer *et al.*, 2010), or the regulation of the *AtPAP1* transgene in tobacco (Liu *et al.*, 2014). More recently, PTRs based on ZFs coupled to an epigenetic effector were developed. This approach allowed the demethylation of the *FWA* gene in *A. thaliana* for controlling flowering time, using the catalytic domain of human TEN-ELEVEN TRANSLOCATION1 (TET1) (Gallego-Bartolomé *et al.*, 2018), a dioxygenase involved in the demethylation of DNA (Chen *et al.*, 2014).

In the last decade, the CRISPR (clustered, regularly interspaced, short, palindromic repeats)/Cas (CRISPR-associated) systems emerged as new versatile programmable effectors. They offer a wide range of applications with high efficiency and specificity, avoiding the main problem that limited previous tools, namely the need to make a new protein for each target (Waryah *et al.*, 2018; Arya *et al.*, 2020). Keeping in mind the same strategy employed in ZFs and TALEs, the endonuclease activity of Cas protein was inactivated through the mutation of specific amino acids in RuvC1 and HNH nuclease domains (L.S. Qi *et al*., 2013). The resulting protein, named ‘dead Cas’ or dCas, can be directed to the target gene promoter and generate a transcriptional response (Larson *et al.*, 2013; Maeder *et al.*, 2013). Compared with ZFs and TALEs, CRISPR-based regulators are much easier to program, requiring only the change of the 20 nucleotide protospacer region in the guide RNA (gRNA) sequence. This approach was described with remarkable results in mammalian cells and other organisms, such as bacteria and fungi (Ho *et al.*, 2020; Mózsik *et al.*, 2021), both for targeted gene activation (also known as the CRISPRa strategy) and for inhibition/repression (CRISPRi), opening up new perspectives for application in plants. The initial strategies to generate PTRs in plants based on CRISPR/dCas9 employed direct fusions of TADs or TRDs to the C-terminus of the dCas9 protein (Fig. 3C). In plants, the initial CRISPRa approaches encompassed the attachment of TAL, VP64, and EDLL regulation domains to the dCas9 structure. The results obtained in targeted transcriptional activation of the *AtPAP1*, *AtFIS2*, and *miR319* genes in *A. thaliana* and the *NbPDS* gene in *N. benthamiana* showed moderate activation rates (Piatek *et al.*, 2015). Following the same strategy, dCas alone or fused to the plant-derived BRD and SRDX domains was employed for transcriptional repression of the *NbPDS* gene and a nopaline synthase promoter driving a luciferase reporter in *N. benthamiana* (Piatek *et al.*, 2015; Vazquez-Vilar *et al.*, 2016), showing the same moderate results.

In subsequent elaborations, new CRISPR PTRs were developed with improved activities by attaching several TADs to the CRISPR/Cas ribonucleoprotein scaffold. Two main strategies were used for this purpose. The SunTag strategy (Fig. 3D) employs a multiepitope fusion peptide that binds chimeric scFV (single-chain variable fragment) nanobodies fused to one or more activation domains (Tanenbaum *et al.*, 2014; Morita *et al.*, 2016). The alternative scRNA strategy (Fig. 3E) introduces RNA aptamers in the gRNA as anchoring sites for additional TADs, which are themselves linked to a viral aptamer-binding domain (MS2) (Fig. 3E). In plants, several dCas9 PTR systems have been developed using these strategies, such as dCasEV2.1 (Selma *et al.*, 2019) or CRISPR–Act2.0 (Lowder *et al.*, 2018). Finally, the CRISPR–Act3.0 strategy (Pan *et al.*, 2021) uses a combination of the scRNA and SunTag strategies, generating a complex formed by the inactivated CRISPR protein, the gRNA, the MS2 protein fused with the SunTag tail, and an scFV antibody fused to activation domains. In addition, this strategy was efficiently transferred to other nucleases, such as dCas12b.

The heterogeneity of target genes and the strong dependence on gRNA efficiency make a comparative analysis of the different available systems a difficult task. However, in general, the second and third generations of CRISPR-based PTRs surpassed—in terms of activation strength, specificity, modularity, and orthogonality—the capacities of most traditional chimeric TFs. Interestingly, CRISPRa tools show unprecedented versatility for multiplex activation. As processor components, this is an interesting capacity in as much as it enables the distribution of a single input signal in several output signalling branches. In other words, multiplexing allows the creation of CRISPR activation ‘programs’ comprising several single-gene activation commands. This can be exploited, among other applications, to selectively activate designated enzymes in a metabolic route (i.e. actuators), thus channelling metabolic fluxes toward specific compounds (Selma *et al.*, 2022b). CRISPRa gRNA programs can be integrated into the plant genome as a part of an SGC or, as recently proposed, they can be exogenously delivered using viral vectors (Khakhar *et al.*, 2021; Selma *et al.*, 2022a). A detailed list of CRISPRa systems developed in plants is provided in Table 1.

**Table 1.** CRISPRa strategies used in plants

Activation strategy	dCas-TAD	Additional TADs	References
Direct fusion of TAD to dCas9	dCas9-EDLL		Piatek *et al.* (2015); Vazquez-Vilar *et al.* (2016)
	dCas9-TV		Li *et al.* (2017)
	dCas9-VPR		
	dCas9-TAL		Piatek *et al.* (2015)
	dCas9-VP64		Lowder *et al.* (2015, 2018); Vazquez-Vilar *et al.* (2016); Li *et al.* (2017)
	dCas9-VP64-EDLL		Lowder *et al.* (2018); Lee *et al.* (2021)
SunTag	dCas9-SunTag	scFv-VP64	Papikian *et al.* (2019)
SAM (aptamers inside the loop)	dCas9-VP64	MS2-p65-HSF1	Park *et al.* (2017)
scRNA (aptamers outside the loop)	dCas9-EDLL	MS2-VPR	Selma *et al.* (2019)
	dCas9-VP64	MS2-EDLL	Lowder *et al.* (2018)
	dCas9-VP64	MS2-VP64	Lowder *et al.* (2018)
SunTag+scRNA	dCas9-VP64-SunTag	MS2-VP64 scFV-2xTAD	Pan *et al.* (2021)

### Synthetic promoters (SPs) as key elements in genetic circuit design

Chimeric TFs as well as PTRs require as an associated resource the availability of synthetic promoters. Such promoters need to be minimally equipped with a core promoter region (aka ‘minimal promoter’) and the appropriate operators, which are the DNA boxes located in a more distal ‘enhancer’ region where TFs will bind.

Core promoters identify the transcription start site (TSS) but promote only basal transcription levels. Until recently, the availability of core promoters for plant engineering was rather limited. The core 35S promoter of cauliflower mosaic virus (CaMV) was widely used with good results (Amack and Antunes, 2020), but multigene engineering requires larger collections to avoid sequence repetition. Cai *et al.* (2020) identified permissive architectures for minimal synthetic plant promoters enabling the computational design of a series of new promoters of different transcriptional strengths. Recently, our understanding of the basal activity of plant core promoters has been substantially extended with the massive analysis of thousands of plant promoters and the subsequent development of computational models to predict and improve promoter strength (Jores *et al.*, 2021), a highly valuable resource for the design of future promoter collections.

The second key elements in SP design are operators, the *cis*-acting elements located in the distal enhancer region, often containing tandem repeats of short DNA sequences (boxes) recognized by the DNA-binding domain of chimeric TFs or a PTR. Typically used operators in plants are those recognized by the LexA (Zuo *et al.*, 2000), GAL4 (Gardner *et al.*, 2009), TetR (Weinmann *et al.*, 1994), or PhiC31 (Vazquez-Vilar *et al.*, 2017) DNA-binding domains. The combination of chimeric TFs and operators is sufficient to create complex regulatory systems for modulating gene expression in plants (Belcher *et al.*, 2020). Furthermore, some operators are directly recognized by proteins performing a dual role of sensors and TFs (e.g. heat shock, glucocorticoid, ethanol, copper, etc.), leading to transcriptional changes in response to an environmental signal, as described in detail in the previous section.

More recently, the customizable binding capacities of PTRs have been exploited to create new collections of synthetic promoters. The strategy behind this approach consists of selecting custom DNA sequences that are known to be recognized efficiently by a certain PTR, and including them as regulatory operators in an enhancer region upstream of a core promoter. Considering that the only functional requirements for the enhancer region are an appropriate distance from the core promoter and the presence of the operator itself, and given the unlimited number of synthetic operators that can be generated using PTRs, this approach can definitively solve the problem of limited availability of promoters for plants. Brückner *et al.* (2015) pioneered this approach by creating 43 promoters containing an 18 base long dTALE-binding site and a core promoter. The new collection was tested successfully in transient assays in *N. benthamiana* (Brückner *et al.*, 2015). More recently, similar approaches have been followed using CRISPR/Cas-based PTRs. The enhanced programmability of CRISPR/Cas brings obvious advantages, leading to the generation of large physical collections of new regulable promoters and an endless number of theoretical designs (Cai *et al.*, 2020; Kar *et al.*, 2022; Moreno-Giménez *et al.*, 2022). Most remarkably, the possibility of combining different *cis*-acting operators in the enhancer region can be used to integrate into a single promoter several positive and negative signals coming from different sensors, thus significantly expanding the operational capabilities of the processors that can be designed in plants.

### Adding complexity to the processor module: Boolean logic gates and memory circuits

**Fig. 4.**
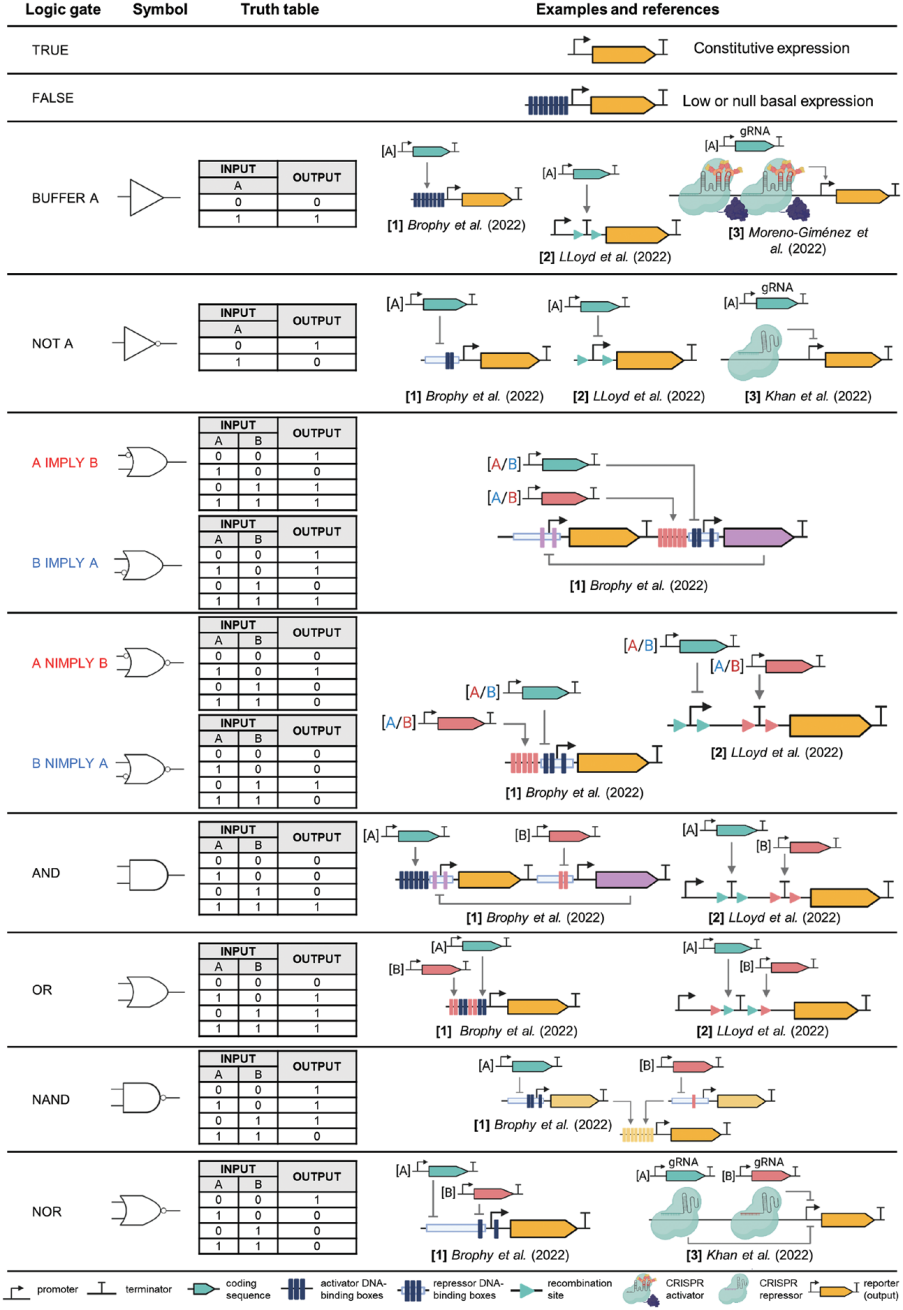
Logic gates. For each logic gate, the symbol, the truth table, and examples of SGCs implemented in plants are displayed. Numbers in brackets indicate examples of logic gates based on [1] chimeric TFs that bind DNA, [2] recombinases that flip or remove promoters or terminators, and [3] CRISPRa or CRISPRi. For IMPLY and NIMPLY gates, the red and blue colour code on the A and B inputs indicates to which of the two alternative logic gates (‘A IMPLY B’ or ‘B IMPLY A’; ‘A NIMPLY B’ or ‘B NIMPLY A’) each input belongs.

Besides activators and repressors operating the simplest identity (activation) and negation (repression) functions, more sophisticated processors have been developed in plants with the introduction of two-input Boolean logic gates. The different logic gates that can be implemented using one or two signal inputs are shown in Fig. 4. These basic operators are very useful in defining spatial–temporal patterns, or to decide outputs from a combination of endogenous and external stimuli. Logic gates are also the building blocks for implementing higher order programs incorporating multiple inputs. Different strategies to produce a full set of synthetic logic gates have been successfully implemented in plants. Using chimeric TFs, Brophy and co-workers created a full set of one- and two-input logic gates in *N. benthamiana* (Brophy *et al.*, 2022). More recently, Khan *et al.* (2022, Preprint) developed a collection of logic gates by combining NOT and NOR gates using an elegant CRISPRi approach and a limited set of synthetic promoters. Other authors have implemented subsets of logic gates with different strategies, including the post-translational genetic control mediated by viral vectors (Cordero *et al.*, 2018), or the use of CRISPRa (Kar *et al.*, 2022; Moreno-Giménez *et al.*, 2022).

A second relevant example of sophisticated processors are memory switches. The ability of a system to retain long-term memory of the singular occurrence of an input signal has many applications, from recording past situations to maintaining a sustained output response once the input signal has vanished. There are different strategies to introduce memory in a gene circuit. In the pioneering work by Gardner *et al.* (2000), two transcriptional repressors acting one against the other were used as the basis to create a bistable toggle switch able to bring memory to the system. Transcriptional toggle switches establishing robust bistable equilibrium states in the cells can be found naturally in plants (Schoof *et al.*, 2000; Cruz-Ramírez *et al.*, 2012), but are difficult to implement synthetically due to the requirements for fine-tune control of transcriptional rates. A relatively simpler alternative consists of introducing covalent changes in the DNA using phage recombinases. Although recombinases had been traditionally used in plants for transgene insertion and removal (Wang *et al.*, 2011), the first transcriptional memory switch in plants was created recently by inserting a promoter region between two reporter genes flanked by PhiC31 integrase sites. The activation of the recombinase prompted the inversion of the promoter, switching OFF the transcription of one reporter gene and turning ON the opposite one (Bernabé-Orts *et al.*, 2020). The system remained in the new ON state in a stable manner once the recombinase was removed. An interesting feature of this new switch is that it is reversible, as it can return to its initial state through the action of a second protein known as the directionality factor. In subsequent elaborations, new recombinase-based memory systems have also been employed to elegantly record single events during root development, showing the suitability of synthetic memory to keep track of cell lineages (Guiziou *et al.*, 2021).

Finally, in a remarkable new approach, Lloyd *et al.* (2022) engineered circuit processors able to both remember and compute. By combining the use of recombinases and plant regulatory elements, the authors created a full set of logic gates that produce sustained outputs in response to a combination of input stimuli. These stabilized responses enable new capabilities for novel plant engineering approaches that require memory-based functions.

## Actuators

The performance of most of the circuit components described in this review has been evaluated by using reporter genes as model actuators. The ratiometric dual luciferase system Luc/Ren is probably the most reliable quantitative system currently in use due to its high sensitivity and large dynamic range (Vazquez-Vilar *et al.*, 2017), although other reporters can provide sufficiently valid outputs in certain contexts. Also, reporters serve to reflect endogenous conditions of the plant, monitoring changes in soil composition, environmental conditions, or the presence of certain pathogens. Beyond reporters, the panoply of actuators that can be assembled to an operator´s transcriptional output is as vast as the repertory of native plants’ biochemical and physiological responses. Beyond that, it can be extended to non-native outputs such as auto-bioluminescence (Mitiouchkina *et al.*, 2020). Perhaps the most obvious actuators are enzymes in biosynthetic pathways in the context of plant metabolic engineering. These pathways can be activated transcriptionally using any of the strategies described above. In an illustrative example, a mini pathway of three enzymes producing an insect pheromone in *N. benthamiana* was activated in response to a copper sensor using a CRISPRa signal distribution strategy with the interplay of new synthetic promoters (Kallam *et al.*, 2023). Other conceivable actuators are ion channels, phosphorylation cascades, chromatin remodellers, defence proteins, etc.

## Perspectives on the assembly of functional circuits in transgenic plants

As reviewed here, the diversity of building blocks from which increasingly complex plant SGCs can be created has expanded considerably in recent years. However, the reports of functional circuits being stably integrated into the genome to generate plants with enhanced abilities are much less abundant, and mostly implemented in model plants such as *A. thaliana*. One likely reason for this is the limitation imposed by genetically modified organism (GMO) regulation, which gives traits resulting from genetic engineering little chance to be deployed realistically in crops. Interestingly though, circuit engineering has important applications in the field of plant biofactories and molecular farming where the prevalent use of non-food crops and the preference for indoor (contained) cultivation makes the application of SGCs more realistic in the short and medium term. Since high yields of recombinant products are frequently associated with a metabolic burden, especially for toxic products, a common goal in molecular farming consists of achieving conditional expression in high-capacity systems, often involving self-replicative units. The engineering of such inducible plant bioproducers is not straightforward. They require tightly regulated circuits that avoid the expression and spread of self-replicative units in the uninduced (OFF) state, releasing all their biosynthetic potential only in the induced (ON) state. Until these systems are developed, *Agrobacterium-*mediated transient expression will most probably continue to be the industry standard, as transient transformation is, after all, nothing more than a rudimentary, albeit expensive, conditional expression system (Kurokawa *et al.*, 2021; Pillet *et al.*, 2022). To date, only a few conditional replicative systems have been reported in stable transgenics. A pioneering mechanism was developed by Werner *et al.* (2011). In this system, tight regulation was achieved by the deconstruction of a tobacco mosaic virus (TMV) replicative system in two modules comprising the replicon, containing the gene of interest, and the cell to cell movement protein. Each module was placed separately under the control of an ethanol-inducible promoter (Werner *et al.*, 2011), reducing background expression. In a similar strategy known as the INPACT system, a geminivirus replicon derived from the ssDNA tobacco yellow dwarf mastrevirus (TYDV) was employed to create a conditional expression system (Dugdale *et al.*, 2013). Here, the gene of interest was split in the replicon vector and only reconstituted in the presence of the TYDV-encoded Rep/RepA proteins. Rep/RepA expression was placed under the control of the AlcA:AlcR gene sensor, which responds to ethanol. Both TMV and TYDV conditional replicative systems were shown to produce massive expression levels (>10% of the total soluble protein) in the ON state with negligible expression in the OFF state.

Apart from the use in plant biofactories, other full SGCs have been assembled in model plants and food crop species, illustrating their potential contribution to agricultural sustainability. A potential application is the anticipation of protective responses to forecasted biotic or abiotic stresses (e.g. harsh climate conditions or plague surveillance) (Goold *et al.*, 2018; Dixon *et al.*, 2021). In a pioneering example, Park *et al.* (2015) engineered an ABA receptor to respond to the agrochemical mandipropamid, thus enabling the use of new agrochemicals to protect against drought stress. Another range of applications for which few examples of plant-integrated circuits already exist is the modification of developmental patterns. Khakhar *et al.* (2018) used synthetic and modular hormone-activated Cas9-based repressors (HACRs) in *A. thaliana* to reprogram development, changing how the hormonal circuitry regulates target genes, as illustrated by the decrease in shoot branching. In another example, Brophy *et al.* engineered a one-input BUFFER gate in *A. thaliana* for the control of root branch density. Arabidopsis lines engineered with different versions of this BUFFER gate linked to the *solitary root* (*slr-1*) gene, a TF gene that represses root branching, showed a range of root branch densities, illustrating how SGCs can be used to influence water and nutrient uptake (Brophy *et al.*, 2022).

Another promising strategy of developmental control consists of linking developmental phase changes to the application of new (preferably environmentally friendly) agrochemicals for which new sensors are developed. Controlling flowering time in response to, for example, ethanol or copper are classic examples of basic circuits developed in model species (Yeoh *et al.*, 2011; Saijo and Nagasawa, 2014). In a remarkable example of this approach applied to crops, Okada *et al.* (2017) engineered rice plants that flower in response to agrochemical spraying. First, non-flowering plants were developed by overexpressing a floral repressor gene to inhibit environmentally induced spontaneous flowering. Later non-flowering plants were co-transformed with a rice florigen gene (Hd3a) expressed under the control of endogenous sensing modules that respond to widely used agrochemicals. For establishing the sensing module, the authors screened promoter sequences capable of inducing transcription using commercially supplied plant activator agrochemicals. In this way, complete circuits implementing a one-input ‘buffer’ function were established. Interestingly, Okada *et al.* (2017) report wide phenotypic variation in flowering responses in the different transgenic rice lines generated, a variation that may be attributable to positional effects or to the genetic backgrounds of the different varieties. These observations illustrate another of the struggles which *in planta* engineering of SGCs is facing, which is the poor reproducibility in stable plants of the quantitative behaviour observed in transient experiments. This is allegedly due to the different epigenome contexts in which transgenes carrying circuit modules are integrated. The current solution to this problem is the screening of many randomly integrated transgenic lines to find those that best reproduce the expected circuit behaviour. In the future, it is expected that the incorporation of targeted genome integration and landing pads strategies will help to circumvent these problems (Altpeter *et al.*, 2016).

## Conclusions

The availability of well-characterized and increasingly elaborated circuit components suggests that soon we will be able to routinely create plants with augmented traits—displaying programmable capacities that go beyond what can be obtained from exploiting natural diversity. However, to reach this point, we still need to learn more about the current engineering limitations, and how to circumvent them. The main questions ahead are those related to genomic integration, and how the genomic context and the epigenomic status affect the quantitative behaviour of a circuit. Understanding how to effectively buffer positional effects, either by inserting buffering DNA elements, applying synthetic epigenetic marks, or by simply designing genome-designated landing pads, will be one of the main lessons to be learned in the coming years. In a related theme, the genomic stability of circuit behaviour also needs to be established. Do circuit components maintain their quantitative behaviour over generations? In that case, will it be possible to combine well-defined components in a modular fashion by, for example, sexual crossing or super-transformation? The experiences obtained so far, not only in plants but also in other systems, seem to indicate that a certain degree of modular behaviour could be expected. This is good news for engineering; however, more systematic analysis of stably integrated circuits will be needed to master this promising bioengineering discipline.
